# N-Terminally Truncated and Pyroglutamate-Modified Aβ Forms Are Measurable in Human Cerebrospinal Fluid and Are Potential Markers of Disease Progression in Alzheimer’s Disease

**DOI:** 10.3389/fnins.2021.708119

**Published:** 2021-07-29

**Authors:** Guido Domingo, Luisa Benussi, Claudia Saraceno, Michela Bertuzzi, Roland Nicsanu, Antonio Longobardi, Sonia Bellini, Alfredo Cagnotto, Mario Salmona, Giuliano Binetti, Roberta Ghidoni

**Affiliations:** ^1^Fondazione IRCCS Istituto Neurologico Carlo Besta, Milan, Italy; ^2^Molecular Markers Laboratory, IRCCS Istituto Centro San Giovanni di Dio Fatebenefratelli, Brescia, Italy; ^3^Department of Molecular Biochemistry and Pharmacology, Istituto di Ricerche Farmacologiche Mario Negri IRCCS, Milan, Italy; ^4^MAC—Memory Clinic, IRCCS Istituto Centro San Giovanni di Dio Fatebenefratelli, Brescia, Italy

**Keywords:** Alzheimer’s disease, mass spectrometry, beta - amyloid peptide, cerebrospinal fluid, pyroglutamate-modified amyloid beta-peptide

## Abstract

Alzheimer’s disease (AD) is a pathology characterized by the accumulation in the brain of intracellular and extracellular amyloid-β (Aβ) aggregates, especially of Aβ1–40 and Aβ1–42 peptides. It is known that N-terminally truncated or modified Aβ forms also exist in AD brains and cerebrospinal fluid (CSF), and they play a key role in the pathogenesis of the disease. Herein, we developed an antibody-free method based on Solid-Phase Extraction and Electrospray Ionization Liquid Chromatography Mass Spectrometry for the identification and quantitation in human CSF of Aβ isoforms. In human CSF, we could detect and quantify a panel of 19 Aβ isoforms, including N-terminally truncated and pyroglutamate-modified forms, never quantified before in CSF. Among these, we identified novel N-terminally truncated Aβ species: four bound to copper and two phosphorylated forms, which were found to be the most common proteoforms in human CSF along with Aβ1–40, Aβ3–40, and AβpE11–42. We tested the newly developed and validated method in a pilot study on CSF from elderly individuals with subjective memory complaints (SMCs, *n* = 9), mild cognitive impairment (MCI, *n* = 18), and AD (*n* = 15); along with Aβ1–42, five N-terminally truncated forms (Aβ11–40, Aβ3–42, AβpE11–42, AβpE3–40, and Aβ4–40 Cu^2+^) are altered in AD/MCI. Thus, we demonstrated that N-terminally truncated and pyroglutamate-modified Aβ can be quantified in human CSF, and five of them, along with Aβ1–42, are potential markers of AD progression. The described method could represent a useful tool for patients’ stratification and monitoring. Moreover, the newly identified Aβ CSF species might represent new potential therapeutic targets.

## Introduction

Alzheimer’s disease (AD) is a pathology characterized by intracellular and extracellular accumulation in the brain of aggregated amyloid-β (Aβ) (Gouras et al., 2000; LaFerla et al., 2007). The “amyloid cascade hypothesis” suggests that the initiating event in AD is an imbalance in the production and clearance of Aβ peptides, leading to the formation of neurotoxic brain Aβ assemblies (Masters et al., 1985; Selkoe, 1991; Hardy and Higgins, 1992). Among these peptides, Aβ1–40 and Aβ1–42 have been the dominant focus of research, but it is well established that N- and C-terminally truncated or modified forms of Aβ also exist in AD brains. Recent evidences suggest that the Aβ-derived fragments generated by secondary cleavages, or the additional amyloid precursor protein-derived fragments, could have a key role in the pathogenesis and the progression of AD (Dunys et al., 2018). Studies revealed that toxic fragments are the N-terminally truncated Aβ peptides, especially the Aβx-42 fragments, probably involved in the very first step of amyloidosis, as suggested by pioneer studies on young Down’s syndrome and preclinical AD brains (Russo et al., 2001; Sergeant et al., 2003; Liu et al., 2006). Aβ4–42 was one of the first N-terminally truncated species to be reported (Masters et al., 1985). This Aβ isoform was found to be highly abundant in familial and sporadic AD brains and aged controls (Portelius et al., 2010; Di Fede et al., 2018). Literature data suggest an important implication in the disease of post-translationally modified forms of N-terminally truncated Aβ derivatives with pyroglutamate at the 3-position (AβpE3) or at the 11-position (AβpE11) (Saido et al., 1995; Dunys et al., 2018). AD comprises different phenotypes characterized by distinct clinical and neuropathological profiles (Lam et al., 2013; Dong et al., 2017). With the use of ultrasensitive and advanced conformation-sensitive techniques, it has been demonstrated that the structure of the different Aβ conformers/assemblies may be responsible for the distinct disease phenotypes (Cohen et al., 2015).

Using a high-resolution mass spectrometry (MS), Wildburger et al. (2017), analyzed intact Aβ peptides from soluble aggregates and insoluble material in brains of AD patients, revealing several N-terminally truncated and post-translationally modified forms. We recently showed that biochemical composition, in terms of Aβ peptides, of amyloid brain aggregates can define two main molecular subtypes of AD: An AD subtype with brain amyloid aggregates enriched in Aβx-42 peptides (including the pyroglutamate-modified Aβ3pE-42 and Aβ11pE-42) and a subtype distinguished by the presence of both Aβx-40 and Aβx-42 peptides, with a prevalence of Aβx-40; these differences affect the physicochemical properties of Aβ assemblies including aggregation kinetics and seeding ability (Di Fede et al., 2018). Interestingly, the preferential accumulation in the brain of specific Aβ fragments is paralleled by a reduction of the very same fragments in the cerebrospinal fluid (CSF) of AD cases (Catania et al., 2015).

To the best of our knowledge, quantitation in CSF of N-terminal pyroglutamate Aβ was never performed before, despite immunohistochemical observations confirming the dominant deposition of the pE3-Aβ peptide in senile plaques (Saido et al., 1995).

Conversely, few N-terminally truncated Aβ peptides (Aβ11-x and Aβ17-x) were quantified, in CSF, using a mid-domain antibody in combination with two commercially available antibodies (4G8 and 6E10) for immunoprecipitation combined with liquid chromatography–tandem MS (Rogeberg et al., 2015). Studies on CSF Aβ proteoforms are almost totally based on an immunoproteomic approach, and most of those assays are time-consuming and expensive and rely on the specific affinity of antibodies. Thus, an antibody-free method able to simultaneously quantify CSF Aβ isoforms is needed.

Herein, we describe an antibody-free method based on Solid-Phase Extraction (SPE) and Ultra-High-Performance Liquid Chromatography (UHPLC)–QqTOF–MS for the simultaneous identification and quantitation in human CSF (hCSF) of 19 Aβ isoforms (Aβ1–42, 1–40, 1–38, and 16 x-40/x-42 N-terminally truncated and post-translationally modified forms—including pyroglutamate forms). The method proposed is validated according to the US FDA guidelines for bioanalytical method validation (Food and Drug Administration of the United States, 2018). The newly developed method was tested on a pilot study on AD patients, mild cognitive impairment (MCI) subjects, and individuals with subjective memory complaints (SMCs) to evaluate its clinical relevance.

## Materials and Methods

### Chemicals and Reagents

Human Aβ synthetic peptides (AβpE11–40, Aβ11–42, AβpE3–40, Aβ3–40, AβpE3–42, Aβ3–42, Aβ4–42, Aβ1–38, Aβ1–40, and Aβ1–42) were purchased from Bachem. Aβ11–40 and nitrogen-15 stable-isotope-labeled Aβ peptides (^15^N53-Aβ1–40) were purchased from r-Peptide. Aβ4–40 was purchased from Primm. AβpE11–42 was synthesized by the Biochemistry and Chemistry Laboratory of the IRCCS Mario Negri Institute (Milan, Italy) (Supplementary Methods and Supplementary Figure 1). All peptide sequences are reported in Supplementary Table 1. Reagents are reported in Supplementary Methods.

### Subjects

Patients underwent clinical and neurological examination at the MAC-Memory Clinic of the IRCCS Fatebenefratelli, Brescia, Italy. Subjects with subjective cognitive impairment, but no objective cognitive impairment (MMSE ≥ 27), were included in the SMCs group; AD patients met the criteria for probable AD (McKhann et al., 1984; Albert et al., 2011; McKhann et al., 2011); MCI subjects met the Petersen et al. (1999) criteria, *n* = 9 converted to AD at follow-up (1.5–6 years). Clinical and demographic characteristics are depicted in Table 1. All patients provided written informed consent. The study was approved by the local ethical committee (Prot. No. 66/2016).

**TABLE 1:** Demographic, clinical, and biochemical characteristic in the three study groups.

	**SMCs (*n* = 9)**	**MCI (*n* = 18)**	**AD (*n* = 15)**	***p***
Gender (% female)	55.6	72.2	66.7	0.69^a^
Age, years	67.0 ± 11.8	72.8 ± 5.3	68.1 ± 9.0	0.16^b^
Education, years	8.9 ± 3.7	7.2 ± 3.2	7.3 ± 3.9	0.47^b^
MMSE	29.0 ± 1.1	26.3 ± 2.5	17.7 ± 7.2	<0.001^b^
CSF Aβ1–42, pg/ml	564.1 ± 183.9	531.5 ± 404.0	374.5 ± 170.8	0.098^c^
CSF tau, pg/ml	274.3 ± 193.7	438.2 ± 174.8	603.2 ± 162.3	<0.001^c^
CSF p-181-tau, pg/ml	51.1 ± 18.5	69.1 ± 34.5	87.3 ± 24.6	<0.01^c^

*Mean ± standard deviation.*

*^a^One-way ANOVA.*

*^b^Chi-square test.*

*^c^Kruskal–Wallis test.*

### Sample Preparation

For all Aβ peptides and for the internal standard (IS; ^15^N53-Aβ1–40), stock solutions were prepared at 1 or 2 mg/ml in DMSO and further diluted in LoBind tubes (Eppendorf) with 0.1% NH_4_OH/ACN (80:20, v/v) to a final concentration of 10 μg/ml and then stored at -80°C. IS solution was diluted to 200 ng/ml in ACN/H_2_O (25:75, v/v) containing 0.5% NH_4_OH (peptide buffer, PB). The working solution for each Aβ peptide was prepared by diluting the initial stock solution, using PB, to have a final concentration of 50 ng/ml for each peptide. Calibration standards and quality control (QC) samples were prepared as described in Supplementary Methods. To test the applicability of this method, 100 μl of hCSF samples was spiked with 10 μl of PB and 10 μl of IS solution (100 ng/ml), vortexed and treated similarly. For copper binding experiments, we added CuSO_4_ (final concentration of 5 mM) to the synthetic peptides. Dephosphorylation was carried out using shrimp alkaline phosphatase (SAP). Before SPE extraction, 1 μl of SAP (1 U/μl) was added to 100 μl of hCSF and incubated at 37°C for 4 h and then inactivated at 65°C for 15 min.

### Solid-Phase Extraction

Spiked hCSF or artificial CSF (aCSF) + BSA was diluted 1:1 with 5 M GuHCl, shaken for 45 min, and diluted 1:3 with 4% H_3_PO_4_ in H_2_O as described in Lin et al. (2017). Each sample was extracted using a μ-elution mixed-mode cation exchange SPE (Oasis PRIME MCX μ-Elution Plate, 30 μm, Waters) method. Samples were first washed with 100 mM ammonium formate with 2% formic acid and then with methanol (MeOH) and eluted with two steps of 5% NH_4_OH in MeOH. The extracted samples were dried and resuspended in H_2_O.

### UHPLC–QqTOF–MS Analysis

Samples were analyzed using a UHPLC–MS system consisting of a Bruker compact QqTOF MS (Bruker Daltonics) and Dionex UltiMate 3000 Rapid Separation LC (Thermo Fisher Scientific) equipped with an Acquity UPLC BEH C18 (300 Å, 2.1 × 150 mm, 1.7 μm) column (Waters). MS parameters were set using microToF 3.4, ESI Compass 1.3, and HyStarPP 3.2 SR4 software (Bruker Daltonics). A 15 μl aliquot of samples was injected into the HPLC column, and elution was performed at a flow rate of 0.2 ml/min using a gradient elution program of 2 min hold at 5% B and 5 to 55% B in 9 min followed by a return to 5% B for a 12 min equilibration. Mobile phase A consisted of 0.3% NH_4_OH in H_2_O while mobile phase B consisted of 90:10 ACN/mobile phase A. The rinse solution of the auto-injector was ACN/H_2_O (50:50, v/v) containing 1% NH_4_OH. The mass range was set from 500 to 2,500 *m*/*z*.

Data processing was done using Data Analysis Software, and peptides were manually quantified using QuantAnalysis 2.2 (Bruker Daltonics).

### Statistical Analysis

A Kolmogorov–Smirnov test was performed in all continuous variables to evaluate the normality distribution. One-way ANOVA with *post hoc* Tukey test, Student’s *t*-test, or Kruskal–Wallis test with Dunn’s correction was used for group comparisons. Categorical variables were performed with a chi-square test. Pearson’s and Spearman’s correlations were employed to test correlations of measures. All analyses were performed by SPSS, and significance was set at 0.05.

## Results

### Method Development and Validation

**FIGURE 1:**
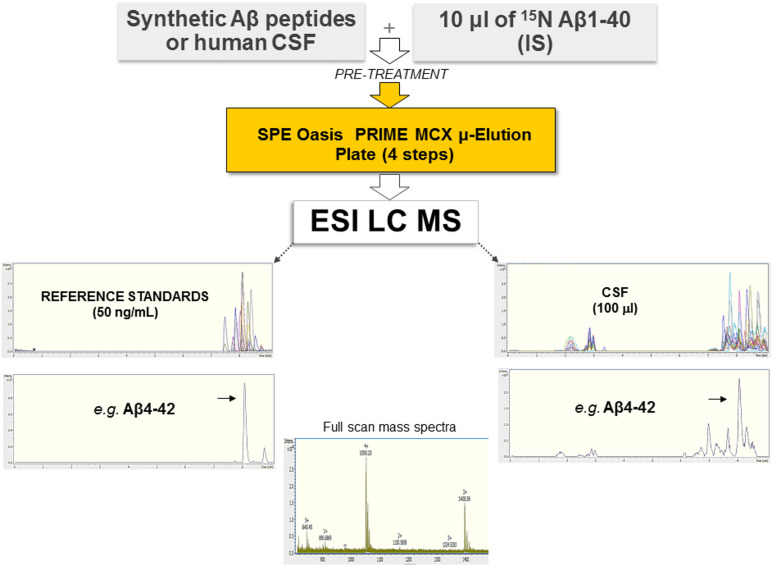
Schematic representation of the method employed, based on SPE and ESI-LC-MS. Synthetic Aβ peptides or hCSF and the isotopically labeled IS were diluted with 5 M GuHCl, shaken at room temperature for 45 min, and then further diluted with 4% H_3_PO_4_ in H_2_O (pre-treatment). Each sample was extracted using a μ-elution mixed-mode cation exchange SPE method. Samples were analyzed using a UHPLC–MS system consisting of a Bruker compact QqTOF mass spectrometer and a Thermo Scientific Dionex UltiMate 3000 Rapid Separation LC equipped with an Acquity UPLC BEH C18 column. Representative spectra of all Aβ peptides as well as of Aβ4–42 are shown.

We developed an antibody-free method based on SPE and ESI-LC-MS for the simultaneous identification and quantitation of Aβ isoforms. A schematic representation of the method is shown in Figure 1. Samples were preprocessed by SPE, and quantification was performed in the positive ion mode using ESI. IS and aCSF were used as a surrogate matrix for calibration curves (Supplementary Figure 2). Chromatographic conditions were optimized to achieve a short run time, acceptable resolution, and symmetrical peak shapes for all the analytes. Chromatograms of all reference standard, performed in positive ion mode and using ESI, are shown in Supplementary Figure 3. Retention time (RT), MS charge states, and theoretical/experimental masses for each Aβ peptide, as well as IS, are summarized in Supplementary Table 3. Due to the low intensity of MS/MS spectra, we chose to base the quantification of each peptide on its MS signal and RT according to Ramagiri and Garofolo (2012), summing multiply charged states (Supplementary Table 3). In hCSF, the identity of each peptide was also confirmed by fragmentation performed in Single Selected Reaction Monitoring (SSRM) mode (data not shown). The method was validated for the fundamental validation parameters following the US FDA guidelines. Over the range of 2.5–75 ng/ml, the peak area ratios were proportional to the concentrations for all peptides. Calibration curves were linear, and the coefficients of the weighted least-squares linear regression ranged from 0.9686 to 0.9990 (Supplementary Figure 2 and Supplementary Table 2). The Lower Limit of Quantification (LLOQ) and Limits of Detection (LODs) are reported in Supplementary Table 4: since analytes had different sensitivities, different LODs were found. Inter- and intra-day precision and accuracy values for the QC samples confirm that the method described has satisfactory accuracy (Supplementary Table 5). After the extraction process, recovery for all analytes was calculated from spiked aCSF samples at Low, Medium, and High QC (LQC, MQC, and HQC, respectively) concentrations. The absolute mean recoveries of all samples ranged between 81.21 and 116.83% (Supplementary Table 6). Among the mean recoveries at LQC, MQC, and HQC levels, the coefficient of variation (CV) percentage was ± 15%, confirming the precision and reproducibility of the method. In aCSF, the matrix effect of CSF constituents was assessed by comparing the responses of the extracted standard QC samples (*n* = 5) with the response of analytes from neat standard samples. All the peak area ratios obtained were within 85 and 115%, demonstrating that no matrix effect occurs. To assess the matrix effect in hCSF, we compared the response of the extracted IS with the response of IS prepared in ACN/H_2_O containing NH_4_OH at equivalent concentrations. Values ranged from 91.66 to 97.84%, indicating that no matrix effect occurs (data not shown). Stability was demonstrated under different conditions, and each analyte was found to be stable in different storage conditions and through three freeze–thaw cycles (Supplementary Table 7). For all Aβ peptides and for the IS, no sample contamination from previous UHPLC analysis (carryover effect) was found.

### Identification of Novel Modified Aβ Species in hCSF Samples

**FIGURE 2:**
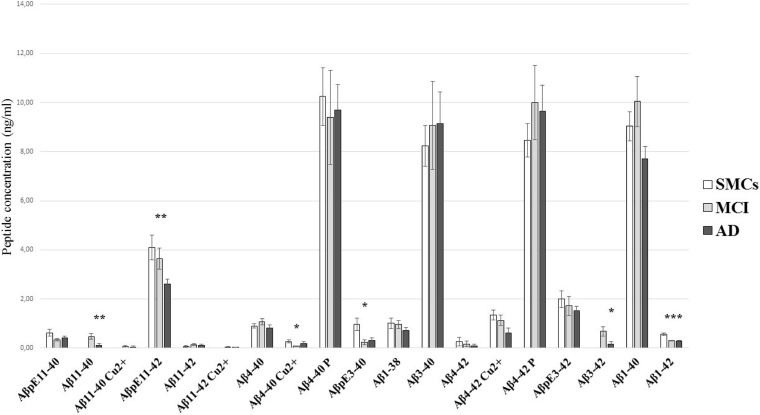
Aβ peptide concentrations in hCSF. Comparison of the Aβ profile in CSF from SMCs, MCI, and AD patients. The quantitation of Aβ peptides by the newly developed ESI-LC-MS method showed substantial differences in Aβ profiles of AD and MCI CSF compared to SMCs. Bars show mean Aβ peptide concentrations (ng/ml) and standard error. Statistically significant differences are reported as *p < 0.05, **p < 0.01, and ***p < 0.001.

We applied the newly developed UHPLC–MS method to clinically relevant hCSF samples, i.e., CSF from MCI subjects (*n* = 18) and AD patients (*n* = 15), and SMC individuals (*n* = 9), as a control group. In hCSF, we could detect and quantify 19 Aβ isoforms, including N-terminally truncated and pyroglutamate-modified forms, some of them present at high concentrations (Table 2 and Figure 2). hCSF Aβ1–42 concentrations by the developed method were correlated with values measured by ELISA (*r* = 0.37, Spearman’s correlation *p* < 0.05, Table 1). To further validate the method, we applied a second complementary method, i.e., the ultrasensitive single-molecule array (Simoa), to measure Aβ1–40: hCSF Aβ1–40 concentrations measured with the two methods were also correlated (*r* = 0.49, *p* < 0.01, Pearson’s correlation, Supplementary Figure 4). Along with all the 13 investigated Aβ isoforms, we detected six additional signal peaks: (i) a peak at *m*/*z* 1,071.5076, where *z* = 3 +, with a mass shift of 20.6263 Da with respect to the experimental mass of Aβ11–40; (ii) a peak at *m*/*z* = 1,133.2333, where *z* = 3 +, with a mass shift of 20.9624 Da with respect to the experimental mass of Aβ11–42; (iii) a signal peak at *m*/*z* = 1,019.3440, where *z* = 4 +, with a mass shift of 15.3291 Da with respect to the experimental mass of Aβ4–40; (iv) a signal peak at *m*/*z* = 1,066.213, where *z* = 4 +, with a mass shift of 16.1702 Da with respect to the experimental mass of Aβ4–42; (v) a peak at *m*/*z* = 1,023.7431, where *z* = 4 +, with a mass shift of 19.7431 Da with respect to the experimental mass of Aβ4–40; and (vi) a signal peak at *m*/*z* = 1,071.0906, where *z* = 4 + with a mass shift of 21.0478 Da with respect to the experimental mass of Aβ4–42 (Supplementary Tables 8, 9). The Aβ11–40/42 and Aβ4–40/42 calculated mass shift, ranging from 61.3164 to 64.6808, could be due to copper binding; in addition, bound and unbound peptides showed the same RT (Supplementary Table 8). To further confirm that the shift of peptide masses observed in CSF was due to a complex with copper, we added CuSO_4_ to HQC. After the incubation with copper, the calculated mass shift of Aβ peptides bound to copper (ranging from + 61.8795 to + 63.9132) is consistent with a single copper-Aβ binding (Supplementary Figure 5). Thus, our study revealed in hCSF a copper binding to Aβ4–42 and, at a lower level, a copper binding to Aβ4–40, Aβ11–40, and Aβ11–42 (Figure 2 and Table 2). In all analyzed CSF, we found that the Aβ4–40 + 19.7431 Da (*z* = 4+) and the Aβ4–42 + 21.0478 Da (*z* = 4+) have the same RT as Aβ4–40/Aβ4–42, suggesting the presence of phosphorylated peptides (Supplementary Table 9). To confirm this, we performed peptide fragmentation in SSRM mode. Of note, both Aβ4–40 and Aβ4–40-P generated an identical fragment (975 *m*/*z*), stripped of phosphate (Supplementary Figure 6); as reported in literature, fragmentation often liberates first the relatively labile phosphate groups (Dephoure et al., 2013). In addition, we performed a dephosphorylation experiment of hCSF using SAP before SPE extraction: In the treated samples, we observed a 54 and a 60% decrease of the Aβ4–40-P/Aβ4–40 and Aβ4–42-P/Aβ4–42 ratios, respectively (Supplementary Figure 7). Thus, we assumed that the Aβ4–40 + 19.7431 Da (*z* = 4 +) and the Aβ4–42 + 21.0478 Da (*z* = 4 +) correspond to the phosphorylated peptides. These identified phosphorylated peptides were found to be two of the most concentrated forms in hCSF elderly subjects, with or without cognitive decline, with a concentration similar to Aβ1–40; along with these peptides, Aβ3–40 and AβpE11–42 forms were among the five most common Aβ peptides in hCSF.

**TABLE 2:** Aβ peptide concentrations in the three study groups.

**Peptides (ng/ml)**	**SMCs (*n* = 9)**	**MCI (*n* = 18)**	**AD (*n* = 15)**	***p***
**AβpE11–40**	0.62 ± 0.14	0.33 ± 0.06	0.41 ± 0.07	0.165^a^
**Aβ11–40**	–	0.47 ± 0.12	0.12 ± 0.06	**0.006** ^c^
**Aβ11–40 Cu^2+^**	–	0.06 ± 0.06	0.05 ± 0.04	0.724^c^
**AβpE11–42**	4.09 ± 0.50	3.64 ± 0.43	2.61 ± 0.19	**0.008** ^a^
**Aβ11–42**	0.07 ± 0.03	0.15 ± 0.05	0.11 ± 0.05	0.631^a^
**Aβ11–42 Cu^2+^**	–	0.03 ± 0.02	0.03 ± 0.02	0.421^a^
**Aβ4–40**	0.90 ± 0.08	1.07 ± 0.12	0.83 ± 0.11	0.298^b^
**Aβ4–40 Cu^2+^**	0.27 ± 0.06	0.07 ± 0.02	0.18 ± 0.07	**0.029** ^a^
**Aβ4–40 P**	10.24 ± 1.18	9.39 ± 1.92	9.70 ± 1.04	0.944^b^
**AβpE3–40**	0.96 ± 0.25	0.24 ± 0.10	0.30 ± 0.12	**0.019** ^a^
**Aβ1–38**	1.01 ± 0.21	0.96 ± 0.16	0.72 ± 0.12	0.511^a^
**Aβ3–40**	8.24 ± 0.83	9.06 ± 1.79	9.15 ± 1.27	0.926^a^
**Aβ4–42**	0.26 ± 0.18	0.17 ± 0.12	0.08 ± 0.08	0.565^a^
**Aβ4–42 Cu^2+^**	1.35 ± 0.20	1.13 ± 0.23	0.60 ± 0.20	0.114^a^
**Aβ4–42 P**	8.46 ± 0.68	10.00 ± 1.52	9.64 ± 1.07	0.750^a^
**AβpE3–42**	2.00 ± 0.34	1.72 ± 0.39	1.52 ± 0.21	0.688^b^
**Aβ3–42**	–	0.68 ± 0.19	0.16 ± 0.11	**0.017** ^c^
**Aβ1–40**	9.03 ± 0.59	10.05 ± 1.02	7.71 ± 0.51	0.149^a^
**Aβ1–42**	0.56 ± 0.05	0.30 ± 0.01	0.29 ± 0.02	**<0.001** ^a^

*Mean ± standard error.*

*^a^Kruskal–Wallis test.*

*^b^One-way ANOVA.*

*^c^Student’s t-test.*

*The bold values are statistically significant.*

### N-Terminally Truncated Aβ Peptides and Pyroglutamate-Modified Aβ Forms Are Altered in MCI and AD

The pilot study on hCSF samples showed that the Aβ1–42 peptide, as expected, was reduced in AD and MCI compared to SMCs; in addition, two N-terminally truncated peptides, Aβ11–40 and Aβ3–42, two pyroglutamate-modified forms, AβpE11–42 and AβpE3–40, and a copper-bound peptide, Aβ4–40 Cu^2+^, were altered in MCI and/or AD (Table 2 and Figure 2). Specifically, AβpE11–42 was significantly reduced in AD compared to SMCs (*p* = 0.012), while AβpE3–40 was reduced in MCI and AD compared to SMCs (*p* = 0.023 and *p* = 0.049, respectively); Aβ11–40 and Aβ3–42 were not detected in SMCs, while they were present in MCI and decreased in AD (*p* = 0.006 and *p* = 0.017, respectively); Aβ4–40 Cu^2+^ was specifically reduced in MCI compared to SMCs (*p* = 0.024).

## Discussion

Literature data suggest an active involvement in AD of N-terminally truncated and post-translationally modified Aβ forms (Saido et al., 1995; Dunys et al., 2018). The unexpected failures of all AD trials targeting the best known Aβ peptides suggest that modified forms, found in AD brains and CSF, may have a pivotal role in AD onset and progression. In line with this hypothesis, recent promising results of a phase 2 trial suggest that treatment with an antibody targeting AβpE3–42 reduces amyloid plaque level as well as cognitive and functional decline in early symptomatic AD patients (Mintun et al., 2021). Herein, we present an antibody-free method based on SPE and UHPLC–QqTOF–MS analysis for Aβ peptide quantitation. This method leads to simultaneous quantitation of Aβ forms, saving time and CSF sample. For the best known Aβ isoforms (Aβ1–42, 1–40, 1–38), the method herein developed showed a sensitivity comparable to previous works (Korecka et al., 2014; Lin et al., 2017) and also for N-terminally truncated and post-translationally modified forms. Using this assay, we demonstrated that several Aβ fragments are measurable in hCSF and that some of them are present at high levels. Many of the Aβ peptides here considered have not been quantified before in CSF. In addition, we described, for the first time in the CSF, the presence of copper–Aβ complexes and phosphorylated Aβ forms. Specifically, we found a copper binding to Aβ4–40/42 and Aβ11–40/42. It has been demonstrated that N-terminally truncated Aβ11–40 and Aβ11–42 have an affinity for Cu^2+^ three orders bigger than Aβ1–40 and Aβ1–42 (Barritt and Viles, 2015). Our study revealed in hCSF a copper binding to the N-terminally truncated forms Aβ4–42, Aβ4–40, Aβ11–40, and Aβ11–42, thus confirming the Cu^2+^ role in AD (Ghidoni et al., 2018). Besides the copper–Aβ complexes, we found the phosphorylated forms of the N-terminally truncated Aβ4–40 and Aβ4–42. The Aβ phosphorylation has been previously described in human and transgenic mouse brains (Kumar et al., 2011, 2013) and seems to influence Aβ aggregation and stability (Kumar et al., 2016; Rezaei-Ghaleh et al., 2016; Barykin et al., 2018). To the best of our knowledge, these phosphorylated N-terminally truncated forms were never described in hCSF. Studies on human brains described a subsequential deposition of Aβ, pyroglutamate-modified Aβ, and phosphorylated Aβ peptides in parenchymal and vascular amyloid deposits: This hierarchical biochemical sequence of Aβ accumulation was associated to AD progression (Rijal Upadhaya et al., 2014; Gerth et al., 2018).

Thus, we investigated whether, in hCSF, the herein quantified Aβ forms might play a role as biomarkers of AD onset and progression. Our pilot study revealed that, along with Aβ1–42, five N-terminally truncated and post-translationally modified N-terminally truncated Aβ peptides, including the Aβ4–40 Cu^2+^, were altered in AD and/or MCI subjects. Specifically, both Aβ11–40 and Aβ3–42 peptides, absent in SMCs, are present in MCI and decreased in AD, reflecting a higher production of N-terminally truncated peptides in early phases, followed by a decrease in AD due to the formation of stable aggregates (Russo et al., 2001; Bouter et al., 2013). AβpE11–42 was found to be reduced in AD compared to SMCs, while AβpE3–40 was reduced in MCI and AD compared to SMCs, indicating that, as suggested by previous studies in the human brain (Rijal Upadhaya et al., 2014; Gerth et al., 2018), the pyroglutamate-modified Aβ forms are potential markers of disease progression. In contrast, the N-terminally truncated phosphorylated Aβ peptides were not altered in MCI or AD: These observations deserve further investigation. Of note, 65% of MCI developed to AD at follow-up: Larger longitudinal studies are needed to investigate whether the quantified Aβ forms can distinguish MCI converting to AD from a stable form.

Even if further investigation, including in cognitively healthy controls, is required to draw major conclusions on the role of post-translationally modified species in AD onset/progression and their putative role as biomarkers, we believe that our method could represent a useful tool for patient stratification and monitoring, also in light of the promising results from the clinical trial targeting pyroglutamate-modified Aβ (Mintun et al., 2021). Moreover, further studies are warranted to clarify the role of the newly identified Aβ CSF species in neurodegeneration, since these species might represent new potential therapeutic targets.

## Data Availability Statement

The raw data supporting the conclusions of this article will be made available by the authors, without undue reservation.

## Ethics Statement

The studies involving human participants were reviewed and approved by Comitato Etico IRCCS San Giovanni di Dio—Fatebenefratelli. The patients/participants provided their written informed consent to participate in this study.

## Author Contributions

RG and GD: conceptualization. GD, MB, SB, and RN: investigation of mass spectrometry and Simoa. AC and MS: investigation of peptide synthesis. GB: clinical investigation. LB: statistical investigation. LB, CS, MB, RN, and AL: data analysis. GD and LB: writing—original draft preparation. RG, CS, MB, AL, AC, MS, SB, and RN: writing—review and editing. All authors: visualization. RG: supervision and funding acquisition.

## Conflict of Interest

The authors declare that the research was conducted in the absence of any commercial or financial relationships that could be construed as a potential conflict of interest.

## Publisher’s Note

All claims expressed in this article are solely those of the authors and do not necessarily represent those of their affiliated organizations, or those of the publisher, the editors and the reviewers. Any product that may be evaluated in this article, or claim that may be made by its manufacturer, is not guaranteed or endorsed by the publisher.
