# Supplementary material for: N-Terminally Truncated and Pyroglutamate-Modified Aβ Forms Are Measurable in Human Cerebrospinal Fluid and Are Potential Markers of Disease Progression in Alzheimer’s Disease
Source: Front Neurosci. 2021 Jul 29;15:708119. doi: 10.3389/fnins.2021.708119 (PMC8358181; doi:10.3389/fnins.2021.708119)
Supplement: Supplementary file 1 [file Data_Sheet_1.PDF]

Supplementary Material

1. Supplementary Methods

1.1 Chemicals and reagents

Human amyloid beta synthetic peptides (AβpE11-40, Aβ11-42, AβpE3-40, Aβ3-40, AβpE3-42, Aβ3-42, Aβ4-42, Aβ1-38, Aβ1-40, Aβ1-42) were purchased from Bachem (Bubendorf, Swiss). Aβ11-40 and Nitrogen-15 stable-isotope labeled amyloid beta peptide (15N53 - Aβ1-40) were purchased from r-Peptide (GA, USA). Aβ4-40 was purchased from Primm (Milan, Italy). All peptide sequences are reported in Table S1. Peptides purity ranges from 81.2% to >98%. The AβpE11-42 peptide (pyroEVHHQKLVFFAEDVGSNKGAIIGLMVGGVVIA) was synthesized by the Biochemistry and Chemistry Laboratory of the IRCCS Mario Negri Institute (Milan, Italy) by solid-phase chemistry using fluorenylmethyloxycarbonyl chloride group (Fmoc) protected amino acid (Sigma–Aldrich, St. Louis, MO, USA) with Initiator+Alstra peptide synthesizer (Biotage, Uppsala, Sweden) at 0.1 mM scale on TGA resin (Novabiochem, Darmstadt, Germany). N-Fmoc deprotection was performed automatically at room temperature by treating the peptide-resin with 20% piperidine in NMP (1-Methyl-2-pyrrolidone) for 3 min followed by another cycle of 10 min and 4 times NMP washed. Amino acid was activated using DIC (*N.N′*-Diisopropylcarbodiimide) and Oxyma pure both at 0.5 mM in NMP. All couplings, using microwave irradiation, were performed at 75°C for 5 min, except for Asp(OtBu). His(Trt). Arg(Pbf) and Boc-L-pyroglutamic-acid couplings which were performed at room temperature for 60 minutes. After each coupling step, the peptide-resin was washed with NMP (4 x 4.5 ml). Peptide was cleaved from the resin with trifluoroacetic acid (TFA)/triisopropylsilane solution (95:5 v/v), precipitated and washed with cold diethyl ether. Purification of AβpE11-42 peptide was carried out with reverse phase HPLC using a semi-preparative C4 column (Symmetry 300, Waters Corporation, Massachusetts, USA) with mobile phases of 0.1% TFA in water (eluent A) and 0.08% TFA in ACN (eluent B), with a linear gradient from 5 up to 100% of eluent B in 60 min.

The peaks were collected and characterized by matrix-assisted laser desorption/ionization-time-of-flight (MALDI-TOF) mass spectrometry with ABI 4800 mass spectrometer, operating in reflector mode (Figure S1). The solutions containing the AβpE11-42 peptide with a purity greater than 95% were finally freeze, dried and the powder stored at -20°C until use.

Artificial cerebrospinal fluid (aCSF) was purchased from Tocris Bioscience (Bristol, UK). Ultra-pure water (resistance >18.2 mΩ), acetonitrile (ACN, gradient grade for liquid chromatography), ammonium hydroxide (NH4OH), dimethyl sulfoxide (DMSO) and guanidine hydrochloride (GuHCl) were all purchased from VWR International Srl (Milan, Italy); shrimp alkaline phosphatase (SAP) was purchased from Roche Applied Science (Germany).

1.2 Samples preparation

Calibration standards and quality control samples were prepared by spiking 10 µl of individual analyte stock solution and 10 µl of IS solution to 100 µl of aCSF containing 4 mg/mL of BSA (bovine serum albumin, Cohn fraction V, heat-shock treated, DNase, RNase and protease free) (Korecka et al., 2014; Lin et al., 2017) except for the double blank samples in which 10 µl of ACN/H2O (25:75, v/v) containing 0.5% NH4OH was added. An eight points standard dilution curve ranging from 2.5 to 75 ng/mL (0, 2.5, 5, 7.5,10, 25, 50, 75 ng/mL) for calibration was prepared. Quality control (QC) samples, independent of the calibration standards, were prepared to evaluate recovery, precision and accuracy of the method at low quality control concentration (LQC; 5 ng/mL), medium quality control concentration (MQC; 25 ng/mL), high quality control concentration HQC; 75 ng/mL) and lower limit of quantification (LLOQ; 2.5 ng/mL). Stability study samples were also prepared in the same procedure at LQC and HQC levels and kept at −20°C until analysis.

2. Supplementary Figures and Tables

Figure S1

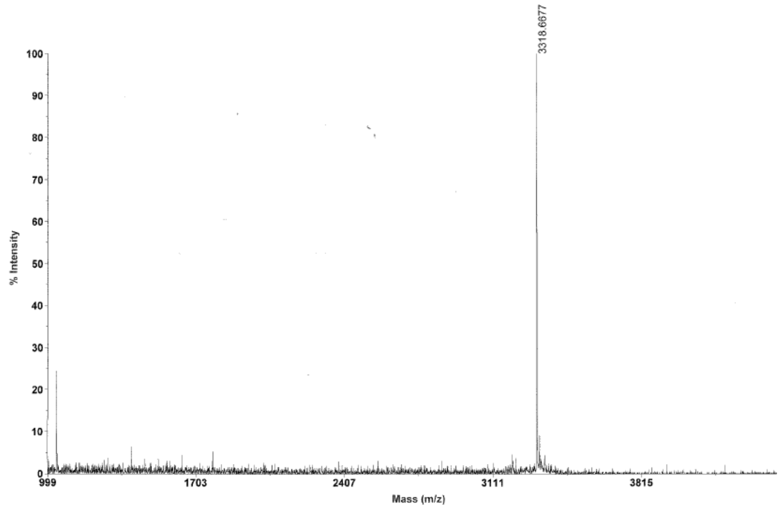

Figure S1. MALDI-TOF analysis of AβpE11-42 peptide after HPLC purification. Spectrum was obtained in reflector mode using the α-Cyano-4-hydroxycinnamic acid as Maldi-Tof matrix.

Table S1

Aminoacid sequences of the quantified Aβ peptides

**Peptide**	**Sequence**
**AβpE11-40**	pEVHHQKLVFFAEDVGSNKGAIIGLMVGGVV
**Aβ11-40**	EVHHQKLVFFAEDVGSNKGAIIGLMVGGVV
**AβpE11-42**	pEVHHQKLVFFAEDVGSNKGAIIGLMVGGVVIA
**Aβ11-42**	EVHHQKLVFFAEDVGSNKGAIIGLMVGGVVIA
**Aβ4-40**	FRHDSGYEVHHQKLVFFAEDVGSNKGAIIGLMVGGVV
**AβpE3-40**	pEFRHDSGYEVHHQKLVFFAEDVGSNKGAIIGLMVGGVV
**Aβ1-38**	DAEFRHDSGYEVHHQKLVFFAEDVGSNKGAIIGLMVGG
**Aβ3-40**	EFRHDSGYEVHHQKLVFFAEDVGSNKGAIIGLMVGGVV
**Aβ4-42**	FRHDSGYEVHHQKLVFFAEDVGSNKGAIIGLMVGGVVIA
**AβpE3-42**	pEFRHDSGYEVHHQKLVFFAEDVGSNKGAIIGLMVGGVVIA
**Aβ3-42**	EFRHDSGYEVHHQKLVFFAEDVGSNKGAIIGLMVGGVVIA
**Aβ1-40**	DAEFRHDSGYEVHHQKLVFFAEDVGSNKGAIIGLMVGGVV
**Aβ1-42**	DAEFRHDSGYEVHHQKLVFFAEDVGSNKGAIIGLMVGGVVIA

Figure S2

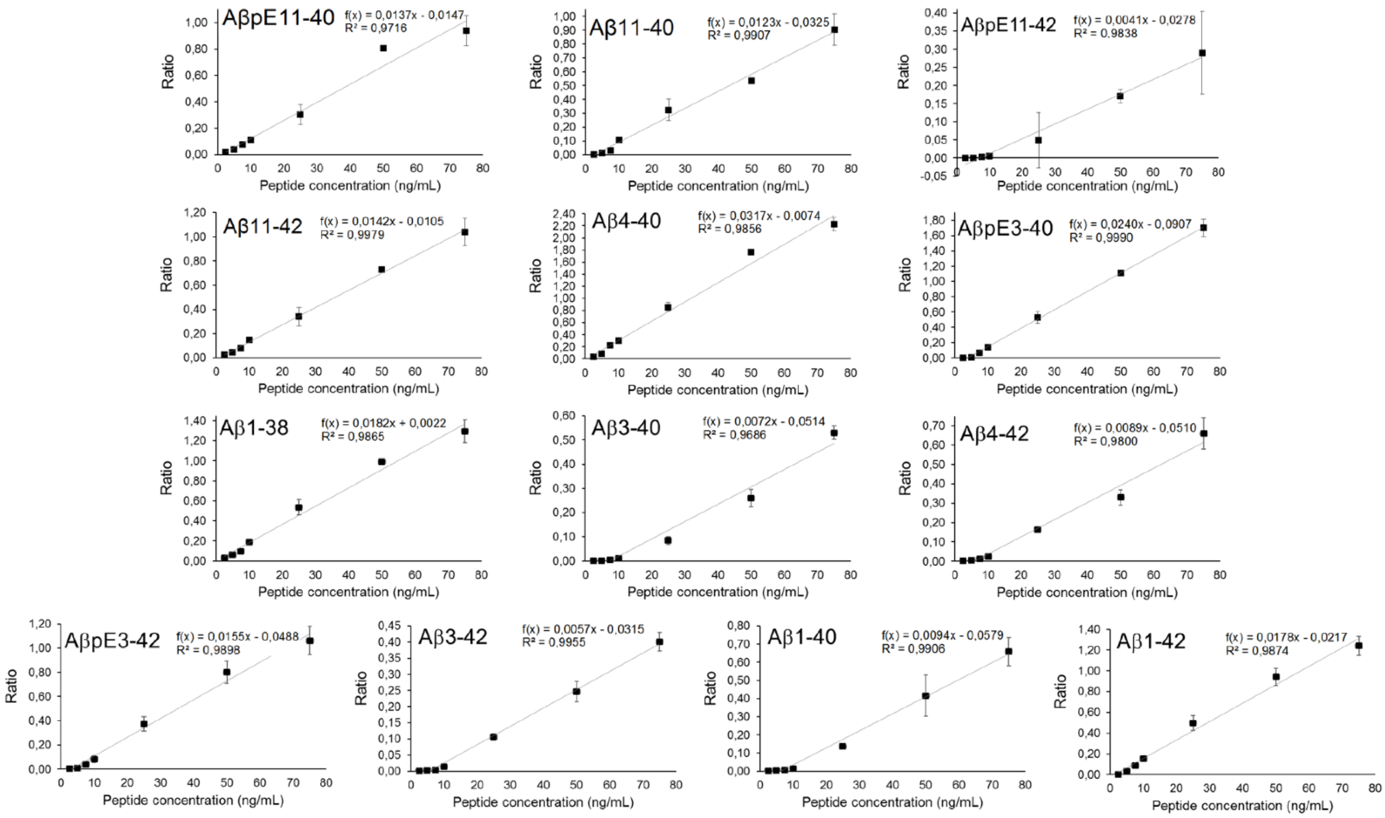

Figure S2. Calibration curves of synthetic Aβ peptides. An eight points standard dilution curve ranging from 2.5 to 75 ng/mL for calibration was prepared of each Aβ peptide. Samples were quantified by the ratio of the peak area of each Aβ peptide to those of the IS (Ratio). Each calibration curve consisted of a blank sample, a zero sample (blank + IS) and seven non-zero concentrations (0, 2.5, 5, 7.5,10, 25, 50, 75 ng/mL). Calibration curves were linear and the coefficients of the weighted least-squares linear regression ranged from 0.9697 to 0.9978.

Table S2

Aβ peptides calibration curves: equation and coefficient of the weighted least-squares linear regressions

Peptide	Equation	R^2
AβpE11-40	F(x)= 0.0137x-0.0147	0.9716
Aβ11-40	F(x)= 0.0123x-0.0325	0.9907
AβpE11-42	F(x)=0.0041x-0.0278	0.9838
Aβ11-42	F(x)= 0.0142x-0.0105	0.9979
Aβ4-40	F(x)=0.0317x-0.0074	0.0986
AβpE3-40	F(x)=0 0.0240x- 0.0907	0.9990
Aβ1-38	F(x)=0.0182x+0.0022	0.9865
Aβ3-40	F(x)=0 0.0072x-0.0514	0.9686
Aβ4-42	F(x)=0.0089x-0.0510	0.9800
AβpE3-42	F(x)= 0.0155x-0.0488	0.9898
Aβ3-42	F(x)=0.0057x- 0.0315	0.9955
Aβ1-40	F(x)= 0.0094x-0.0579	0.9906
Aβ1-42	F(x)=0.0178x-0.0217	0.9874

Figure S3

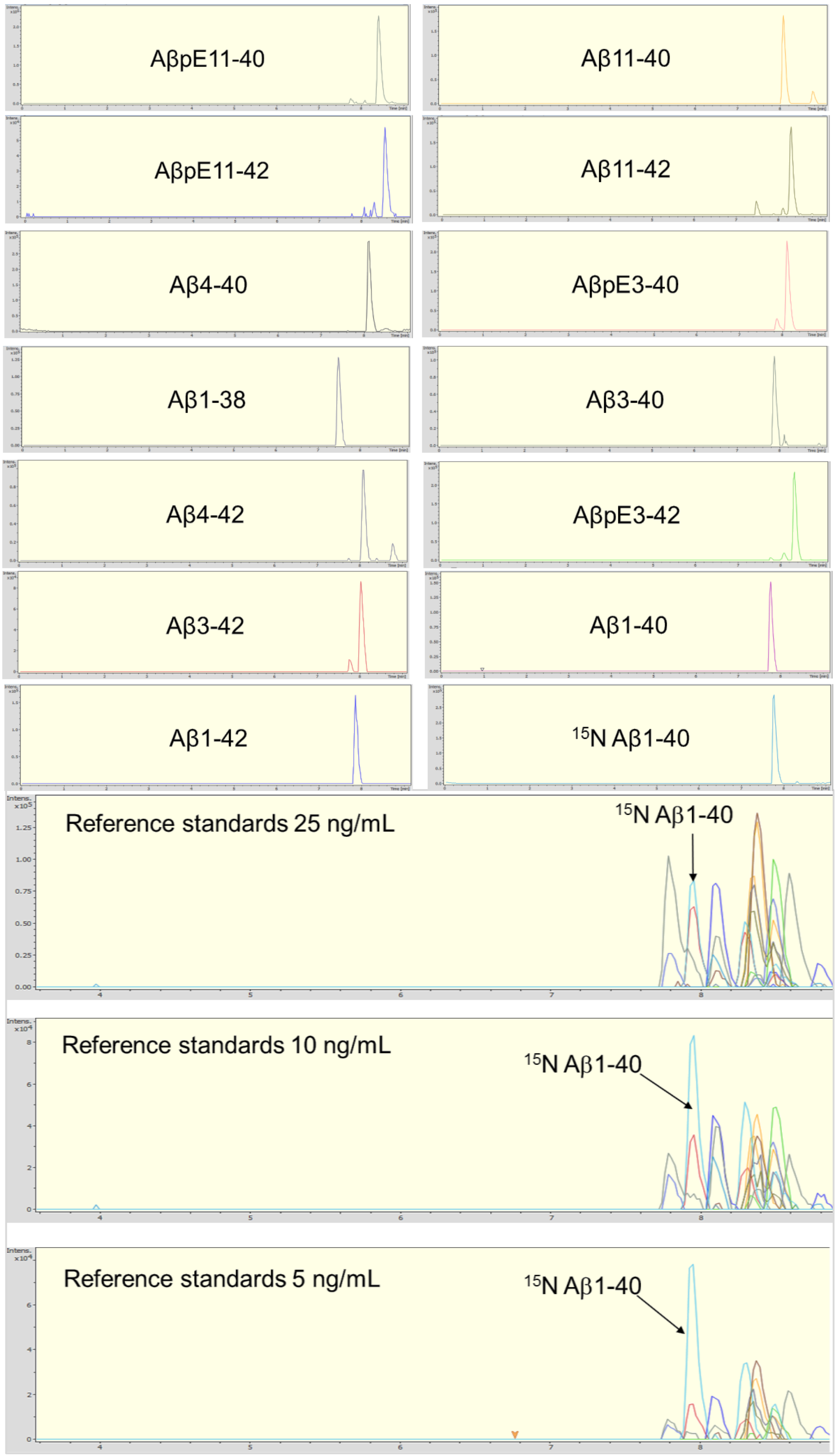

Figure S3 Representative chromatograms of all reference standards (single synthetic Aβ peptides and combined peptides at different concentrations) performed in positive ion mode using ESI.

Table S3

Retention times (RT), MS charge states, theoretical and experimental masses for each Aβ peptide and isotopically labelled IS.

Peptide	RT (min)	Theoretical Mass[1] [M + H]+ (Da)	Precursor ion charge state (m/z)	#1 Theoretical Mass[1] (Da)	#1 Experimental Mass (Da)	Mass error (ppm)	Precursor ion charge state (m/z)	#2 Theoretical Mass[1] (Da)	#2 Experimental Mass (Da)	Mass error (ppm)
AβpE11-40	8.65	3133.6503	$[M + 3H]3^{+}$	1045.2216	1045.2099	11.19	$[M + 2H]2^{+}$	1567.3288	1567.3297	-0.57
Aβ11-40	8.30	3150.6769	$[M + 3H]3^{+}$	1050.8971	1050.8813	15.03	$[M + 2H]2^{+}$	1575.8421	1575.845	-1.84
AβpE11-42	8.75	3317.7715	$[M + 3H]3^{+}$	1106.5953	1106.5863	8.13	$[M + 2H]2^{+}$	1659.3894	1659.4132	-14.34
Aβ 11-42	8.45	3334.7980	$[M + 3H]3^{+}$	1112.2709	1112.2569	12.59	$[M + 4H]4^{+}$	834.455	834.4605	-6.59
Aβ4-40	8.25	4013.0491	$[M + 4H]4^{+}$	1004.0177	1004.0149	2.79	$[M + 3H]3^{+}$	1338.3545	1338.2904	47.9
AβpE3-40	8.40	4125.0651	$[M + 4H]4^{+}$	1032.0217	1032.0137	7.75	$[M + 3H]3^{+}$	1375.6932	1375.6975	-3.13
Aβ1-38	7.70	4130.0189	$[M + 4H]4^{+}$	1033.2602	1033.2716	-11.03	$[M + 3H]3^{+}$	1377.3445	1377.3493	-3.48
Aβ3-40	8.10	4142.0916	$[M + 4H]4^{+}$	1036.2784	1036.2768	1.54	$[M + 3H]3^{+}$	1381.3687	1381.3748	-4.42
Aβ4-42	8.40	4197.1702	$[M + 4H]4^{+}$	1050.0480	1050.0428	4.95	$[M + 3H]3^{+}$	1399.7283	1399.6944	24.22
AβpE3-42	8.50	4309.1863	$[M + 4H]4^{+}$	1078.0520	1078.0433	8.07	$[M + 3H]3^{+}$	1347.0669	1347.0686	-1.26
Aβ3-42	8.30	4326.2128	$[M + 4H]4^{+}$	1082.3087	1082.3095	-0.74	$[M + 3H]3^{+}$	1442.7425	1442.7425	-0.14
Aβ1-40	8.00	4328.1557	$[M + 4H]4^{+}$	1082.7944	1082.7961	-1.57	$[M + 3H]3^{+}$	1443.3901	1443.4037	-9.42
Aβ1-42	8.05	4512.2769	$[M + 4H]4^{+}$	1128.8247	1128.8300	-4.70	$[M + 3H]3^{+}$	1504.7638	1504.7552	5.72
Aβ1-40 (^{15}N)	8.00	4380.9986	$[M + 4H]4^{+}$	1096.0051	1096.0100	-4.47	$[M + 3H]3^{+}$	1461.0044	1461.0209	-11.29

** Theoretical masses were calculate using ProteinProspector. v 5.24.0. MS-Product. proteomics tools for mining sequence databases*

Table S4

Limits of detection and limits of quantifications of Aβ peptides

Peptide	LOD (ng/mL)	LLOQ (ng/mL)
AβpE11-40	0.50	1.00
Aβ11-40	0.50	1.00
AβpE11-42	1.00	2.50
Aβ 11-42	0.50	1.00
Aβ4-40	0.50	1.00
AβpE3-40	1.00	2.50
Aβ1-38	0.50	1.00
Aβ3-40	1.00	2.50
Aβ4-42	1.00	2.50
AβpE3-42	0.50	1.00
Aβ3-42	1.00	2.50
Aβ1-40	0.50	1.00
Aβ1-42	0.50	1.00

Table S5

Intra- and inter-day accuracy and precision values for the QC samples

Peptide	Precision (% RSD)								Accuracy (% Bias)							
	LLOQ (2.5 ng/mL)		LQC (5 ng/mL)		MQC (25 ng/mL)		HQC (75 ng/mL)		LLOQ (2.5 ng/mL)		LQC (5 ng/mL)		MQC (25 ng/mL)		HQC (75 ng/mL)	
	Inter-day	Intra-day	Inter-day	Intra-day	Inter-day	Intra-day	Inter-day	Intra-day	Inter-day	Intra-day	Inter-day	Intra-day	Inter-day	Intra-day	Inter-day	Intra-day
**AβpE11-40**	17.99	14.11	12.40	13.94	2.32	2.39	1.42	0.64	-14.55	-13.84	-3.50	-5.62	0.18	0.78	2.59	3.36
**Aβ11-40**	13.85	19.87	4.62	5.09	7.12	7.25	5.17	5.41	-12.13	-9.47	-6.94	-7.74	2.25	0.13	-4.15	-5.43
**AβpE11-42**	18.76	19.00	6.49	1.27	5.26	5.99	0.96	1.07	18.02	17.37	8.05	12.04	-0.09	-0.68	0.19	0.34
**Aβ 11-42**	10.87	11.86	5.55	5.96	5.83	5.90	1.81	1.08	-19.99	-18.54	-12.38	-13.49	2.93	1.17	-3.29	-4.16
**Aβ4-40**	19.32	17.65	8.26	9.44	10.99	9.73	3.49	3.50	-17.44	-17.40	-12.73	-13.56	3.88	-0.60	1.08	2.05
**AβpE3-40**	8.65	16.11	2.04	2.34	3.72	4.29	1.99	1.85	-19.76	-13.86	-14.88	-14.77	4.38	4.42	-2.39	-3.06
**Aβ1-38**	16.76	19.86	6.87	7.48	2.25	2.09	1.67	0.98	-18.63	-17.53	-13.97	-14.14	4.95	5.75	2.37	3.23
**Aβ3-40**	8.89	15.44	5.78	5.36	8.21	5.72	1.20	1.29	-19.00	-16.76	-13.74	-12.09	-3.62	-0.06	2.11	2.35
**Aβ4-42**	17.01	18.77	11.52	13.29	4.36	4.88	3.37	3.42	16.03	18.13	9.54	4.84	-0.63	-0.06	2.08	1.11
**AβpE3-42**	9.05	13.76	7.53	8.35	7.50	7.25	1.35	1.38	-18.48	16.42	-13.61	-14.81	-4.17	-6.57	-1.18	-1.54
**Aβ3-42**	8.76	10.48	2.62	0.41	7.83	6.08	2.67	2.75	9.04	13.64	0.09	1.58	-0.27	2.97	3.14	3.84
**Aβ1-40**	10.97	12.75	2.80	2.76	3.94	1.98	3.62	3.94	11.10	17.54	9.00	9.90	5.22	7.36	-1.51	-2.23
**Aβ1-42**	17.64	15.55	2.22	2.28	5.42	5.08	2.08	1.78	19.55	18.55	11.86	12.50	2.85	4.67	2.87	3.69

Table S6

Extraction recoveries of synthetic Aβ peptides spiked in aCSF

Extraction recoveries									
	LQC (5 ng/mL)			MQC (25 ng/mL)			HQC (75 ng/mL)		
**Peptide**	Mean	SD	%CV	Mean	SD	%CV	Mean	SD	%CV
**AβpE11-40**	85.52	4.85	-7.73	91.26	8.31	-8.52	85.67	3.51	-12.37
**Aβ11-40**	81.22	14.16	-11.08	99.71	10.75	-1.03	85.00	0.59	-14.48
**AβpE11-42**	103.64	1.57	8.70	91.94	6.73	-11.45	101.58	9.76	0.97
**Aβ 11-42**	84.58	2.73	-11.20	91.07	5.90	-8.28	93.49	0.36	-8.23
**Aβ4-40**	106.31	10.71	8.65	101.64	6.31	3.96	99.62	2.43	-0.65
**AβpE3-40**	107.25	8.55	5.43	98.37	21.94	3.11	105.36	1.34	3.44
**Aβ1-38**	98.83	7.64	-5.72	100.87	5.63	4.43	92.50	1.23	-6.76
**Aβ3-40**	94.83	2.91	-12.88	103.25	4.93	-5.90	97.96	4.32	0.70
**Aβ4-42**	77.64	1.53	-11.82	101.18	6.89	-1.80	90.19	5.50	-5.92
**AβpE3-42**	73.36	3.66	-14.75	97.39	11.26	-8.88	86.22	2.58	-15.12
**Aβ3-42**	116.84	4.18	15.25	119.03	4.21	9.67	104.12	5.47	5.99
**Aβ1-40**	96.70	2.13	4.95	82.67	1.60	-13.55	98.74	3.68	0.02
**Aβ1-42**	94.54	1.21	4.54	110.43	2.55	8.02	97.88	8.85	-1.13

Table S7

Stability recovery and bias of Aβ peptides under different conditions

Peptide	**(20°C 6 h)**						**Freeze-thaw (-80°C x3)**						**Auto-sampler (4°C 24h)**					
	LQC			HQC			LQC			HQC			LQC			HQC		
	Mean conc. (ng/mL)	Bias (%)	% Stability recoveries	Mean conc (ng/mL)	Bias (%)	% Stability recoveries	Mean conc. (ng/mL)	Bias (%)	% Stability recoveries	Mean conc. (ng/mL)	Bias (%)	% Stability recoveries	Mean conc. (ng/mL)	Bias (%)	% Stability recoveries	Mean conc. (ng/mL)	Bias (%)	% Stability recoveries
**AβpE11-40**	5.43	8.63	88.84	72.52	-3.31	106.10	4.25	-14.92	113.42	71.89	-4.14	107.02	4.73	-5.31	101.91	65.91	-12.12	116.74
**Aβ11-40**	5.55	11.07	83.78	75.10	0.13	95.72	4.80	-4.06	97.00	69.04	-7.95	104.13	4.33	-13.41	107.47	76.87	2.49	93.52
**AβpE11-42**	5.53	10.66	97.64	72.01	-3.98	104.35	5.91	18.24	91.38	71.18	-5.09	105.57	5.43	8.61	99.48	76.73	2.31	97.94
**Aβ 11-42**	3.98	-20.43	110.13	73.57	-1.90	98.58	4.21	-15.85	104.13	66.22	-11.71	109.53	4.70	-5.96	93.18	68.45	-8.74	105.97
**Aβ4-40**	4.03	-19.48	108.38	80.26	7.02	94.45	4.03	-19.49	108.40	68.52	-8.64	110.63	4.43	-11.44	98.53	73.49	-2.02	103.16
**AβpE3-40**	4.21	-15.81	101.11	80.73	7.64	90.68	4.15	-16.97	102.52	63.09	-15.87	116.03	4.02	-19.53	105.78	63.85	-14.87	114.66
**Aβ1-38**	4.34	-13.21	100.41	78.19	4.25	98.19	4.98	-0.41	87.51	78.31	4.41	98.04	4.02	-19.59	108.38	79.65	6.20	96.39
**Aβ3-40**	4.06	-18.88	106.34	68.44	-8.75	111.90	4.16	-16.86	103.75	79.09	5.45	96.83	4.17	-16.63	103.46	70.96	-5.38	107.91
**Aβ4-42**	5.68	13.51	93.24	77.51	3.35	98.77	5.81	16.27	91.02	70.58	-5.90	108.48	4.20	-15.95	125.91	70.23	-6.36	109.02
**AβpE3-42**	4.15	-17.05	104.15	70.68	-5.76	104.86	4.17	-16.58	103.56	66.89	-10.81	110.79	4.41	-11.84	97.99	67.15	-10.47	110.37
**Aβ3-42**	5.05	0.99	99.10	76.57	2.10	101.03	4.97	-0.55	100.64	67.25	-10.33	115.03	5.00	-0.06	100.14	77.48	3.30	99.85
**Aβ1-40**	5.20	3.91	104.90	65.11	-13.18	113.44	5.24	4.72	104.10	72.24	-3.68	102.25	5.22	4.47	104.34	73.15	-2.46	100.97
**Aβ1-42**	5.39	7.72	103.85	75.12	0.16	102.71	5.26	5.11	106.42	73.54	-1.94	104.91	5.25	5.08	106.45	76.30	1.74	101.12

Table S8

Retention times (RT) and experimental masses of Aβ11-40/42 and Aβ4-40/42 bound or not to Cu^{2+}

Peptide	RT (min)	Precursor ion charge state (m/z)	#1 Theoretical Mass* (Da) Copper unbound	#1 Experimental Mass (Da) Copper unbound	#1 Experimental Mass (Da) With shift	Observed Δ Mass shift (Da)	Calculated Aβ Δ Mass shift (Da)
**Aβ11-40**	8.30	$[M + 3H]3^{+}$	1050.8971	1050.8813	1071.5076	20.6263	61.8789
**Aβ11-42**	8.45	$[M + 3H]3^{+}$	1112.2709	1112.2569	1133.2333	20.9624	62.8872
**Aβ4-40**	8.25	$[M + 4H]4^{+}$	1004.0177	1004.0149	1019.3440	15.3291	61.3164
**Aβ4-42**	8.40	$[M + 4H]4^{+}$	1050.0480	1050.0428	1066.2130	16.1702	64.6808

*Theoretical masses were calculated using ProteinProspector, v 5.24.0, MS-Product

Figure S4

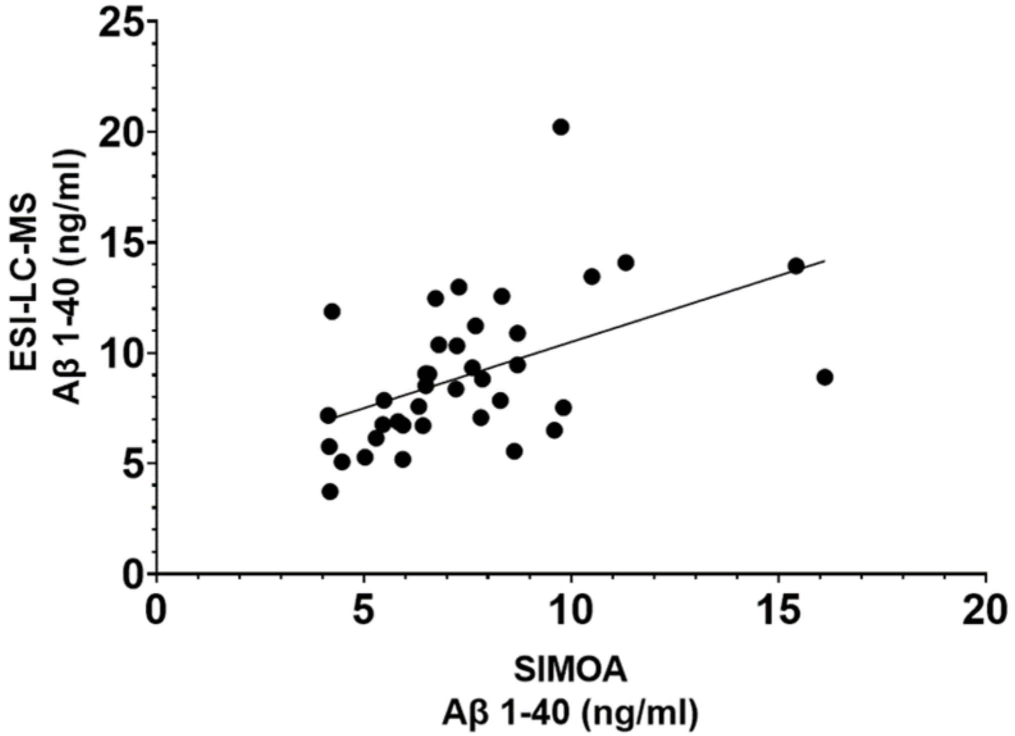

Figure S4. Correlation of Aβ1-40 concentrations (ng/ml) measured by the ESI-LC-MS method and by Simoa. hCSF Aβ1-40 levels were measured by the Simoa™Aβ40 Advantage Kit using an SR-X analyzer (Quanterix, Lexington, USA): SMCs (n=7): 6.65±0.78ng/ml; MCI (n=18): 8.34±3.34ng/ml; AD (n=15): 6.57±1.93ng/ml; p=0.121. hCSF Aβ1-40 concentrations measured by ESI-LC-MS and Simoa were correlated (r=0.49, p<0.01 Pearson's test).

Figure S5

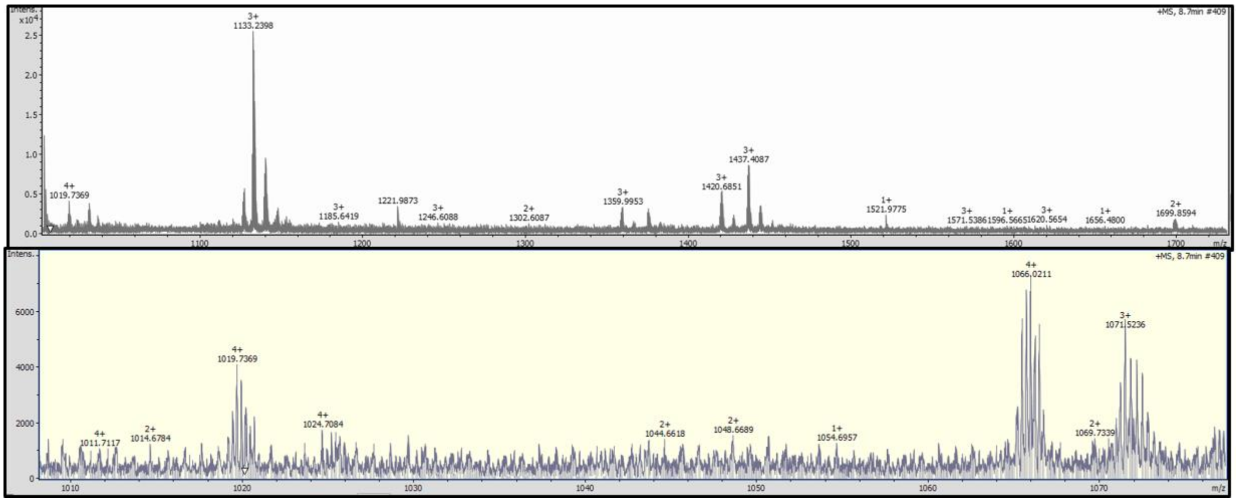

Figure S5. A representative full scan mass spectrum of Aβ peptides incubated with $CuSO_4$ (5 mM). The m/z of 1019.73, 1133.23, 1066.02 and 1071.52 are respectively the product ion spectra of the copper adducts with Aβ4-40, Aβ11-42, Aβ4-42 and Aβ11-40.

Table S9

Retention times (RT). MS charge states and experimental masses in human CSF for Aβ4-40. Aβ4-42 and their phosphorylated forms

**Peptide**	RT (min)	Precursor ion charge state (m/z)	#1 Experimental Mass (Da) Unphosphorylated	#1 Experimental Mass (Da) Phosphorylated	Observed Δ Mass shift (Da)	Calculated Aβ Δ Mass shift (Da)
**Aβ4-40**	8.25	$[M + 4H]4^{+}$	1004.0149	1023.7580	19.7431	78.9724
**Aβ4-42**	8.40	$[M + 4H]4^{+}$	1050.0428	1071.0906	21.0478	84.1912

Figure S6

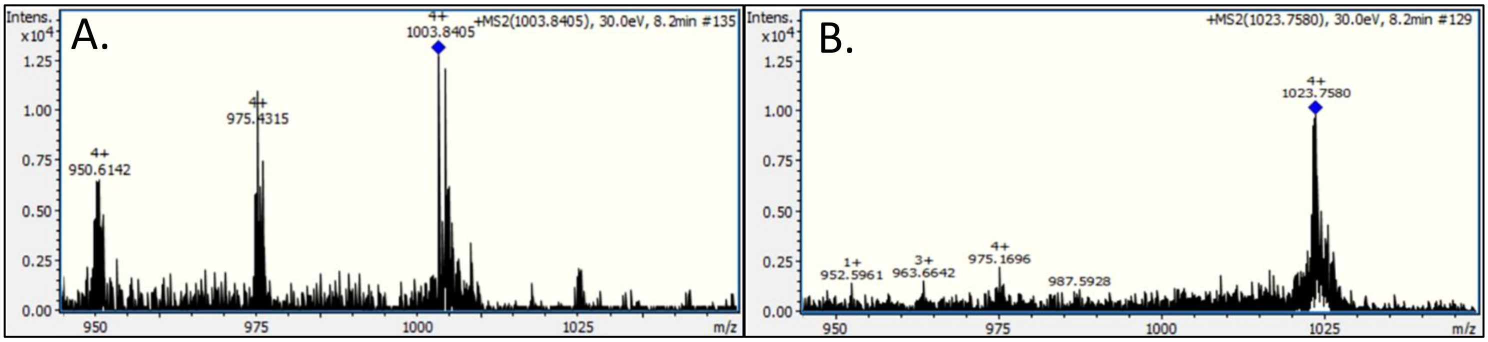

Figure S6. Representative MS/MS spectra of **(A)** Aβ4-40 peptide and **(B)** phosphorylated Aβ4-40 peptide (Aβ4-40P) in human CSF; SRM transitions are: 1003>975 m/z for the Aβ4-40, 1023>975 for the Aβ4-40P.

Figure S7

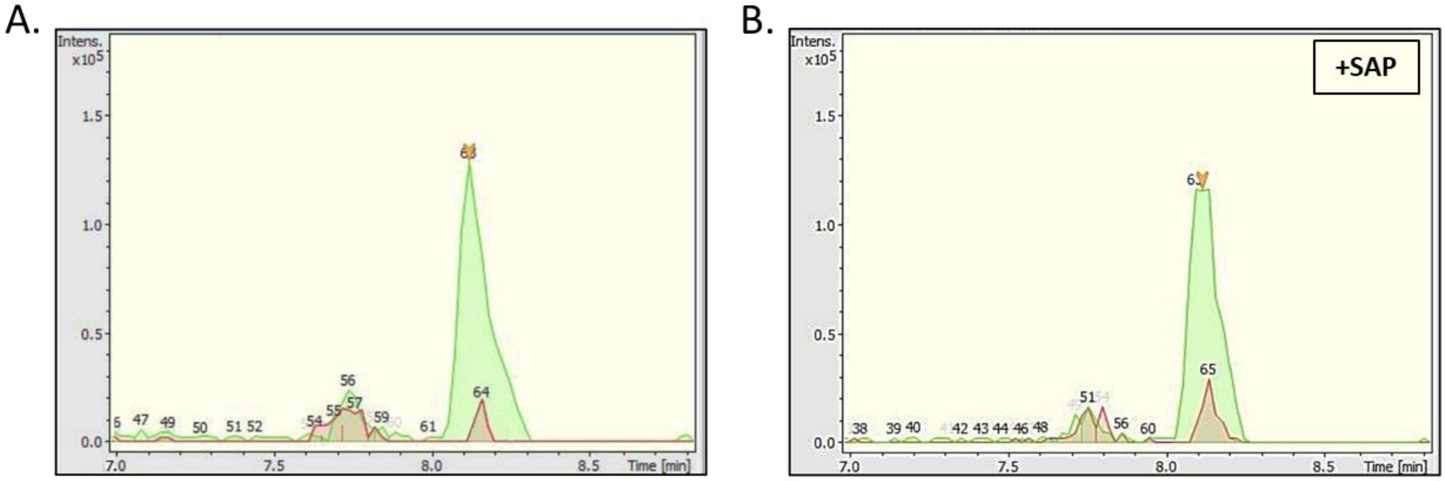

Figure S7. Representative UHPLC-MS chromatograms of Aβ4-40P (green) and Aβ4-40 (red) in hCSF **(A)** untreated and **(B)** treated with SAP (dephosphorylation experiment). Peaks'areas: (A) Aβ4-40-P = 808.825, Aβ4-40 = 56.594; Aβ4-42-P = 90.704, Aβ4-42 = 20.502; (B): Aβ4-40-P = 810.350, Aβ4-40 = 123.815; Aβ4-42-P = 73.088, Aβ4-42 = 40.992.
